# Effect of MDI on the Mechanical Properties of Fibers in Poly(lactic acid)/Poly(butylene succinate) Blends During Melt Spinning

**DOI:** 10.3390/polym18010073

**Published:** 2025-12-26

**Authors:** Ye-dam Jeong, Hyun Je Cho, Min Jae Seo, Jongwon Kim

**Affiliations:** 1 Korea Textile Machinery Convergence Research Institute, Gyeongsan 38542, Republic of Korea; ydjeong@kotmi.re.kr; 2 Department of Fiber System Engineering, Yeungnam University, Gyeongsan 38541, Republic of Korea; karon27@naver.com (H.J.C.); smjid0330@naver.com (M.J.S.)

**Keywords:** poly(lactic acid), poly(butylene succinate), miscibility, melt spinning, blend

## Abstract

In this study, the properties of poly(lactic acid) (PLA)/poly(butylene succinate) (PBS) blends were analyzed according to the PBS content during the manufacture of the blend. However, the inherent immiscibility between PLA and PBS often leads to phase separation and limited mechanical performance, particularly in melt-spun fiber applications, which restrict their practical use. To increase the miscibility of the PLA/PBS blend, methylene diphenyl diisocyanate (MDI) was added up to 0.8 wt.%, and the characteristics were analyzed via thermogravimetric analysis, differential scanning calorimetry, viscosity measurements, dynamic mechanical analysis, and Fourier-transform infrared spectroscopy. As the PBS content in the blend increased, the thermal stability, viscosity, elastic properties, and glass transition temperature decreased. In contrast, as the MDI content in the PLA/PBS blend increased, the thermal stability, viscosity, elastic properties, and glass transition temperature increased. The results revealed that the miscibility of the PLA/PBS blend increased as the MDI content in the blend increased. Additionally, the tensile strength and elongation of the PLA/PBS blend fibers manufactured through melt spinning were analyzed. While the tensile strength decreased as the PBS content increased, the tensile strength and elongation considerably improved as the MDI content in the blend increased. Specifically, the tensile strength of the PLA/PBS blend fibers increased from 2.55 to 2.99 gf/de, corresponding to an improvement of approximately 17%, while the elongation at break increased from 22.48% to 41.64%, representing an enhancement of approximately 85% with increasing MDI content.

## 1. Introduction

Recently, global eco-friendly trends have gained significant attention, including carbon neutrality, which reduces greenhouse gas emissions and eliminates remaining greenhouse gases, and the concept of a sustainable circular economy, which maximizes resource utilization, reduces waste, and recycles it to create a virtuous cycle of resources. If the current pattern of plastic consumption continues, it is predicted that by 2050, 1.1 billion tons of plastic will be produced annually using 20% of global oil, causing serious problems due to plastic waste. To address these concerns, research is focused on replacing existing nondegradable materials with biodegradable materials. Consequently, interest in bioplastics is rapidly increasing worldwide [1].

Bioplastics are bio-based polymers manufactured using biological resources such as plant biomass. Among bioplastics, biodegradable polymers undergo chemical and physical changes as they decompose under certain conditions after use, ultimately being converted into water, carbon dioxide, nitrogen, and biomass. This transition is caused by environmental factors such as light, heat, and humidity, as well as the action of microorganisms such as fungi and bacteria [2,3,4,5]. While regular plastics take approximately 500 years to decompose naturally, biodegradable plastics can decompose in less than a year, making them a key bioplastic to solve the plastic waste problem. Biodegradable polymers are mainly composed of ester, amide, and ether structures, and can be polymerized using petroleum-based raw materials and biomass-based raw materials. However, unlike nondegradable polymers, biodegradable polymers have disadvantages such as relatively high manufacturing costs and deterioration of physical properties due to decomposition before use. Therefore, to completely replace nondegradable polymers, methods to improve basic properties and durability must be investigated.

Poly(lactic acid) (PLA) is a biodegradable material obtained from corn starch and sugarcane. It is an aliphatic polyester-based biodegradable polymer and a thermoplastic aliphatic polyester with ester bonds in the repeating unit and hydroxyl and carboxyl groups in the terminal ends, enabling hydrolysis and microbial decomposition [6,7]. Additionally, unlike other biodegradable polymers, it can be easily mass-produced and is considered an eco-friendly material that can replace commercial plastic. PLA has the advantage of producing considerably low greenhouse gas emissions than nondegradable polymers, with carbon dioxide emissions from raw materials to production (cradle-to-factory gate) of 0.62 kg CO_2_–eq per kg in its life-cycle assessment [8]. Accordingly, it is used in various applications, including automobile interior and exterior materials, disposable containers, and packaging containers. However, PLA has shortcomings such as low melting strength, low thermal stability, and high brittleness; therefore, research is ongoing to improve its properties through blending with other polymers [9,10]. In particular, blends with flexible polymers such as poly(butyl acrylate) (PBA), poly((butylene succinate)-co-adipate) (PBSA), poly(butylene succinate) (PBS), and poly((butylene adipate)-co-terephthalate) (PBAT) have been reported [11,12,13,14]. Among these, studies on blending PLA with PBS have been reported mainly for its use as a polymer to improve the brittleness, crystallinity, and mechanical properties [15,16,17,18,19].

Among various flexible biodegradable polymers, PBS has been considered one of the most suitable blending components for PLA because of its aliphatic polyester structure, good ductility, and relatively low melting temperature, which allows facile melt processing. In addition, PLA and PBS share similar ester-based chemical structures, providing potential sites for intermolecular interactions or reactive compatibilization through terminal functional groups. Compared to other flexible polymers such as PBAT or PBA, PBS offers balanced mechanical flexibility while maintaining biodegradability and process stability, making it particularly attractive for fiber-processing applications.

Previous studies on PLA/PBS blends have mainly focused on improving the brittleness and crystallization behavior of PLA by PBS incorporation [15,16,17,18,19]. However, most reports have consistently shown that PLA and PBS are intrinsically immiscible, resulting in phase-separated morphologies and deterioration of mechanical properties, especially under melt-processing conditions. To overcome this limitation, various approaches such as the addition of plasticizers, reactive additives, and copolymeric compatibilizers have been proposed to enhance interfacial adhesion and suppress phase separation in PLA/PBS blends [20,21,22,23,24]. Despite these efforts, systematic investigations on the effect of reactive compatibilization on the properties of PLA/PBS blends during melt spinning and subsequent fiber performance remain limited. In contrast to most previous studies that mainly focused on bulk or film properties of PLA/PBS blends, this study emphasizes reactive compatibilization using 4,4′-diphenylmethane diisocyanate (MDI) in melt-spun PLA/PBS fibers and systematically investigates its effects on fiber-specific thermal, mechanical, and viscoelastic properties.

A polymer blend is a material that has improved properties, compared to each individual polymer, achieved by mixing two or more polymers with different chemical structures and properties. In practical applications, the use of single polymers is gradually decreasing, while the application of polymer blends with improved properties tailored to specific requirements is increasing [25]. Polymer blends can be divided into miscible systems, which can be mixed at the molecular level; immiscible systems, which cannot be mixed; and partially miscible systems, which mix only to a limited extent. In polymer blends, compatibility is rare, and most systems exhibit either incompatibility or partial compatibility is observed. This is due to the difficulty of achieving uniform interactions between molecular chains throughout the material and the difference in interfacial tension caused by the different chemical structures between polymer chains. These factors cause phase separation between polymers and deteriorate the properties of the blend. Recently, many studies have been conducted to develop methods to improve the miscibility of incompatible polymer blends [26,27], and, in particular, active research is being conducted to improve the interfacial bonding between each polymer blend using miscibilizing agents [28,29]. PBS is also a polymer that is incompatible with PLA, and phase separation can result in deterioration of mechanical properties. Therefore, research has been conducted to address this limitation by adding plasticizers, reactive additives, and copolymers [20,21,22,23,24].

To improve the miscibility of immiscible or partially miscible polymer blends, various compatibilization strategies have been proposed, including the use of reactive additives. Among them, diisocyanate-based compounds have been widely used as reactive compatibilizers or chain extenders in polyester-based polymer systems. These additives can react with hydroxyl or carboxyl end groups of polyesters, leading to chain extension, branching, or the formation of covalent bonds at the interface between different polymer phases. As a result, interfacial adhesion can be enhanced and phase separation can be effectively suppressed.

In particular, MDI has been reported to improve the compatibility and mechanical properties of biodegradable polyester blends by promoting interfacial coupling through urethane or amide bond formation [30]. Therefore, the incorporation of MDI is considered an effective approach to enhance the miscibility of PLA/PBS blends during melt processing.

This study aims to improve the brittleness of PLA by blending it with an appropriate amount of PBS. Because PLA and PBS have poor miscibility, an appropriate amount of MDI was added to mitigate phase separation, resulting in a PLA/PBS blend. This blend was melt-spun to produce PLA/PBS fibers that compensate for the disadvantages of each polymer, and their characteristics were systematically investigated.

## 2. Experimental

### 2.1. Materials

PLA used in this study was Q/ABFPLA 004-2021 from BBCA BIOCHEMICAL, and poly(butylene succinate) (PBS) used was BG5000-M from ANKOR BIOPLASTICS. Table 1 presents detailed specifications of PLA and PBS. 4,4′-methylene diphenyl diisocynate (MDI) used as a miscibilizing agent in the blend was DAEJUNG’s CAS no. 101-68-8 product. The melt-spinning machine used was A JIN MACHINERY COMPANY’s SE-200 product.

### 2.2. Preparation of PLA/PBS Blends and PLA/PBS Blend Fibers

**Figure 1:**
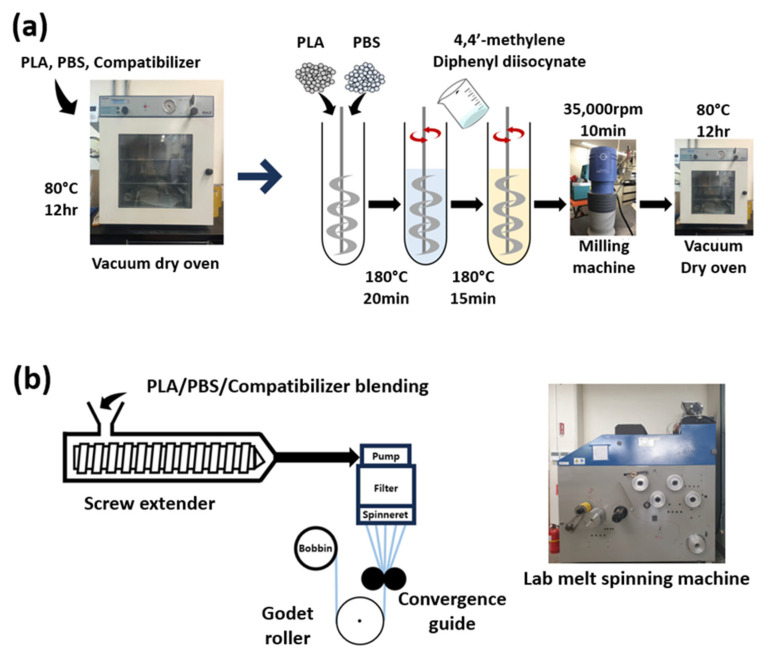
PLA/PBS blending fiber manufacturing schematic diagram. (**a**) The manufacturing of PLA/PBS blends, and (**b**) the manufacturing of PLA/PBS fiber using a melt-spinning machine.

The specimens were manufactured through two processes: blend manufacturing according to the blend ratio of PLA/PBS and the MDI content, and PLA/PBS fiber manufacturing through melt spinning. The PLA/PBS blend ratio during blend manufacturing is presented in Table 2, and the MDI content for PLA/PBS is presented in Table 3. Before manufacturing the PLA/PBS blend, PLA, PBS, and MDI were vacuum dried at 80 °C for 12 h. The vacuum-dried sample was melted in an oil bath at 180 °C for 20 min by varying the PBS content to PLA at 0, 10, 20, 30, and 40 wt.%, and then MDI was added at 0, 0.2, 0.4, 0.6, and 0.8 wt.% and stirred at 180 °C for 15 min. The manufactured sample was milled into powder form at 35,000 rpm for 10 min using a milling machine (IKA A11 Basic Analytical Mill, IKA-Werke GmbH & Co., Staufen im breisgau, Germany), and then vacuum dried at 80 °C for 12 h. Blended resins manufactured according to the blend ratio of PLA/PBS and the MDI content were spun through a melt-spinning machine. The melt-spinning conditions were a spinning temperature of 200 °C, a winding speed of 100 m/min, and a mass flow rate of 0.55 g/min. The specifications included a spinneret size of 1 mm and 10 spinneret holes. Figure 1 is a schematic diagram of the PLA/PBS specimen manufacturing process.

### 2.3. Measurement of PLA/PBS Blends and PLA/PBS Blend Fibers

To analyze the thermal properties of the PLA/PBS blend resin, a thermogravimetric analyzer (TG-DTA, SDT Q600, Ta Instruments, New Castle, DE, USA) and a differential scanning calorimeter (DSC, Q200, SDT Q600, TA Instruments, New Castle, DE, USA) were used to measure the decomposition temperature and melting point according to the blend ratio of PLA/PBS and the MDI content. The viscosity of the blend resin was measured according to temperature using a rheometer (MCR 302e, Anton Paar, Graz, Austria). To analyze the miscibility of the PLA/PBS blend resin, a dynamic mechanical analyzer (DMA, Q800, TA Instruments, New Castle, DE, USA) was used to measure the deformation that appears when stress is repeatedly applied to specimens manufactured according to the blend ratio of PLA/PBS and the MDI content, and viscoelasticity analysis was performed. The chemical structure of the specimen was analyzed using FT-IR (Spectrum 100, Perkin Elmer, Waltham, MA, USA) measurements. The fibers made by spinning the resin manufactured according to the blend ratio of PLA/PBS and the MDI content were measured for tensile strength and elongation at break using a universal testing machine (OTT-05, Oriental Co., Siheung-si, Republic of Korea). The elongation at break was calculated using Equation (1):

(1) $$Elongation\left(\%\right)=\frac{E_{1}-E}{E}\times 100,$$

where *E* denotes the original length (mm) and *E_1_* denotes length at break (mm).

To confirm the phase separation phenomenon in the manufactured fiber, the cross-section was examined using a scanning electron microscope (SEM, S-4800, Hitachi, Tokyo, Japan).

## 3. Results and Discussions

### 3.1. Characteristics of Blends Manufactured According to the PLA/PBS Blend Ratio

#### 3.1.1. TGA According to the PLA/PBS Blend Ratio

**Figure 2:**
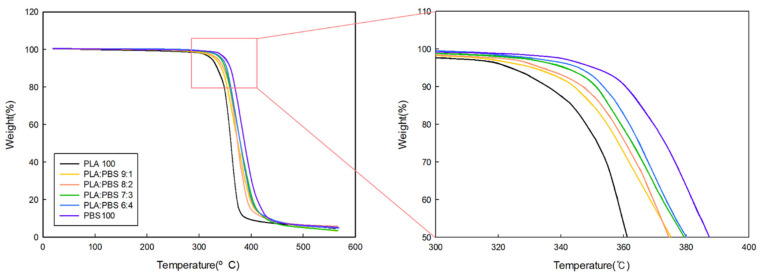
TGA curves according to the PLA/PBS blend ratio.

Thermogravimetric analysis (TGA) was performed to verify the thermal decomposition characteristics of the blends manufactured according to the PLA/PBS blend ration as a function of temperature. Figure 2 shows the TGA graph of the blends as a function of the PLA/PBS ratio. PLA was found to begin decomposing first around 300 °C, while PBS decomposed around 370 °C. As the PBS content increased, the temperature at which decomposition began also increased, indicating that thermal stability increased as the PBS content within the blend increased. To quantitatively support this tendency, the thermal decomposition onset temperatures obtained from the TGA curves are summarized in Table 4. As the PBS content increased, the onset temperature gradually increased, which can be attributed to the inherently higher thermal stability of PBS compared to PLA.

#### 3.1.2. DSC Analysis According to PLA/PBS Blend Ratio

**Figure 3:**
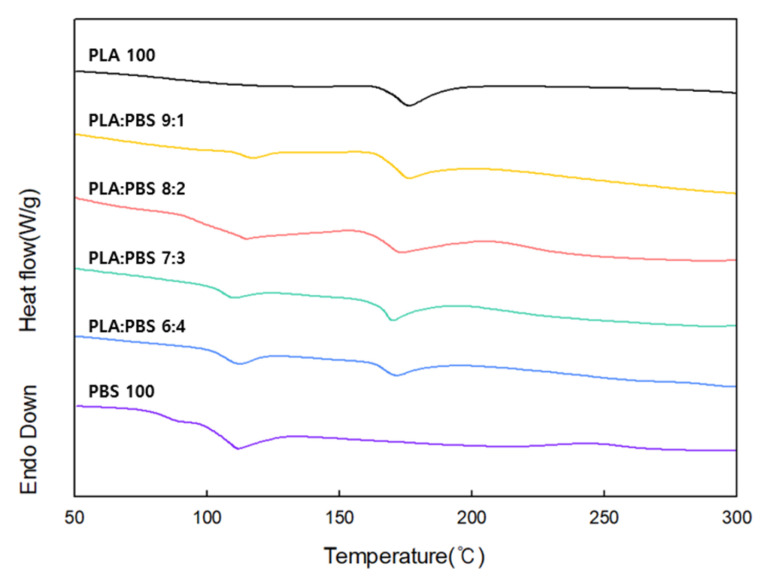
DSC curves according to the PLA/PBS blend ratio.

Figure 3 shows the DSC graph according to the PLA/PBS blend ratio. The endothermic peak of DSC was measured to confirm the melting temperature according to the PLA/PBS blend ratio. The endothermic peaks for PLA and PBS were observed at 176 °C and 114 °C, respectively. In the case of the blend, it was confirmed that two endothermic peaks appeared at approximately 176 °C and 114 °C, regardless of the PBS content. This separation of peaks is believed to be attributed to the poor miscibility between PLA and PBS.

#### 3.1.3. Rheological Analysis According to the PLA/PBS Blend Ratio

**Figure 4:**
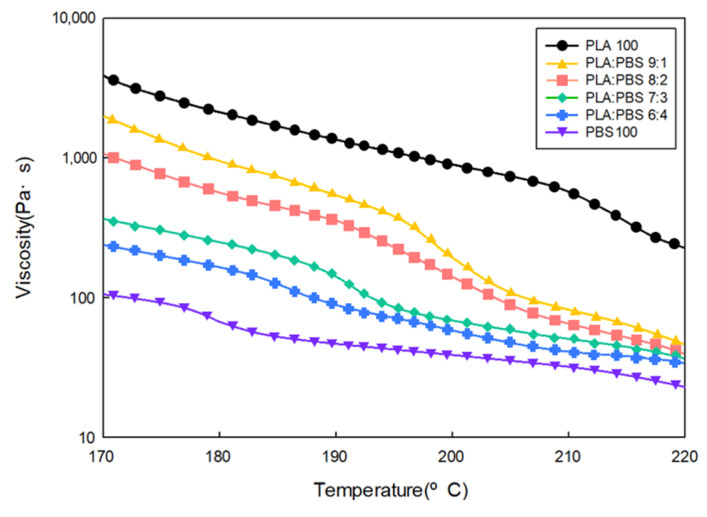
Viscosity according to the PLA/PBS blend ratio.

Figure 4 illustrates the viscosity of the blends as a function of temperature, showing the rheological characteristics according to the PLA/PBS blend ratio. The viscosity of all specimens decreased as the temperature increased. PLA exhibited the highest viscosity, and the viscosity decreased as the PBS content increased because PBS has a lower viscosity than PLA.

#### 3.1.4. Viscoelastic Behavior Analysis According to the PLA/PBS Blend Ratio

**Figure 5:**
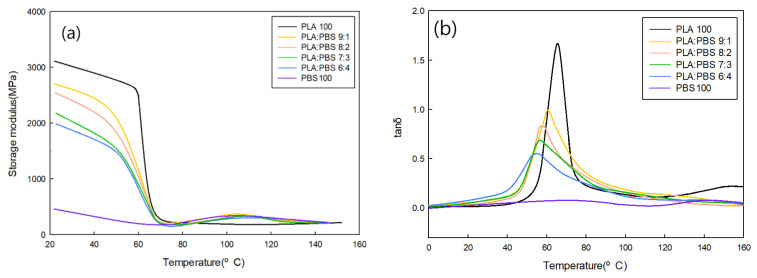
(**a**) Storage modulus and (**b**) tan δ according to PLA/PBS blend ratio.

To analyze the viscoelastic behavior according to the PLA/PBS blend ratio, deformation under repeated stress was measured using DMA. Figure 5a displays the storage modulus curve of the specimen, and the elastic properties of the specimen were confirmed through the storage modulus. The storage modulus of PLA was the highest, and as the PBS content increased, the storage modulus of the blend decreased. This is because the blended specimens exhibit poor miscibility. Figure 5b displays the tan δ curve of the specimens, and the glass transition temperature of the specimen was confirmed through the tan δ peak. The glass transition temperature of PLA is 65 °C, and it decreased as the PBS content in the blend increased. This is because the miscibility decreases as PBS is added to the blend, and the tan δ peak shifts toward PBS, which has a lower glass transition temperature than PLA. Melt-spinning conditions for PLA/PBS blends were established through a characteristic analysis of the PLA/PBS blend ratio. Melt spinning was performed at PLA/PBS blend ratios of 10:0, 9:1, 8:2, 7:3, and 6:4.

### 3.2. Characteristics of Fibers Manufactured According to the PLA/PBS Blend Ratio

#### Tensile Strength and Elongation at Break of Fiber According to PLA/PBS Blend Ratio

**Figure 6:**
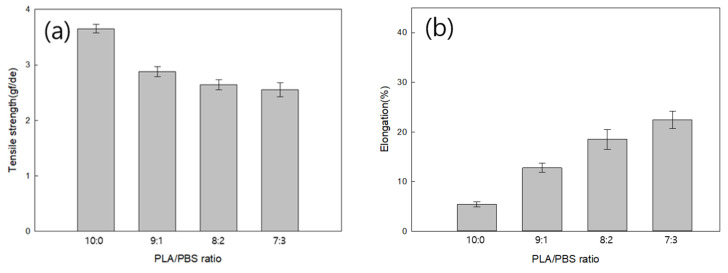
Tensile strength (**a**) and elongation at break (**b**) of blend fibers according to PLA/PBS blend ratio.

Figure 6a shows the tensile strength of fibers manufactured with PLA/PBS blend ratios. The tensile strength of neat-PLA fiber was 3.65 gf/de, while that of the blend fiber with a PLA:PBS ratio of 7:3 decreased by approximately 30.14% to 2.55 gf/de. This is because the tensile strength of the blend fiber decreased due to the addition of PBS, which has a relatively low tensile strength, compared to PLA. When the PLA:PBS ratio was 6:4, fiber breakage frequently occurred during melt spinning. This is because PLA and PBS have poor miscibility. Figure 6b displays a graph of the elongation at break of fibers manufactured according to the PLA/PBS blend ratio. The elongation at break of neat-PLA fiber was 5.44%, while that of the blend fiber with a PLA:PBS ratio of 7:3 increased by approximately 313.24% to 22.48%. As the PBS content in the blend increased, the elongation increased, because the high elongation of PBS compensated for the brittleness of PLA.

### 3.3. Characteristics of PLA/PBS Blends According to MDI Content

After melt-spinning PLA/PBS blends at different blend ratios, fibers in the form of filaments could be produced up to a blend ratio of 7:3, but it was impossible at a blend ratio of 6:4. Accordingly, the characteristics of blends with PLA:PBS ratios of 7:3 and 6:4 were analyzed by varying the MDI content. A preliminary experiment was conducted to establish the content ratio of MDI to be added to the PLA/PBS blend. Consequently, fibers could not be produced when more than 0.8 wt.% was added. Therefore, specimens were prepared by adding 0, 0.2, 0.4, 0.6, and 0.8 wt.% of MDI.

#### 3.3.1. TGA of PLA/PBS Blends According to MDI Content

**Figure 7:**
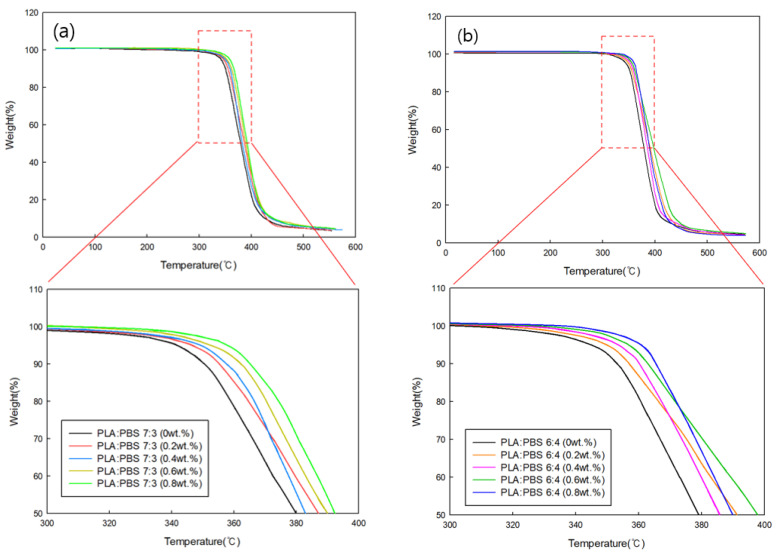
TGA curves of (**a**) PLA/PBS (7:3) and (**b**) PLA/PBS (6:4) blends according to MDI content.

TGA was performed to determine the thermal decomposition characteristics of PLA/PBS blends with varying MDI content. Figure 7a shows a TGA graph for a PLA:PBS blend ratio of 7:3 at varying MDI content, and Figure 7b shows a TGA graph for a PLA:PBS blend ratio of 6:4 at varying MDI content. In both conditions, the decomposition onset temperature increased as the MDI content increased, compared to the blend without MDI. This is because the thermal stability of the blend specimen increased as the miscibility of PLA and PBS improved due to MDI additions. To quantitatively support this tendency, the thermal decomposition onset temperatures obtained from the TGA curves are summarized in Table 5. For both PLA:PBS blend ratios (7:3 and 6:4), the onset temperature gradually increased with increasing MDI content. This result indicates that the incorporation of MDI enhances the thermal stability of the blends, which can be attributed to improved interfacial interactions and restricted chain mobility resulting from enhanced miscibility between PLA and PBS.

#### 3.3.2. DSC Analysis of PLA/PBS Blends According to MDI Content

**Figure 8:**
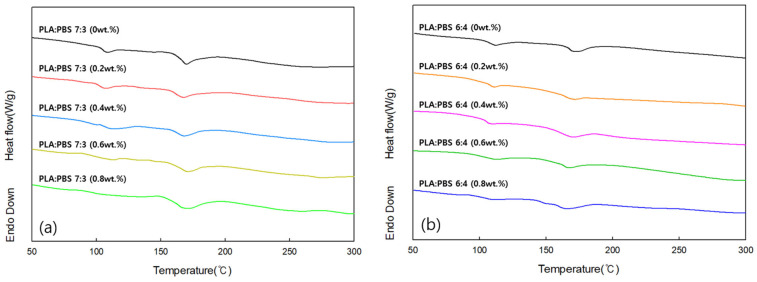
DSC curves of (**a**) PLA/PBS (7:3) and (**b**) PLA/PBS (6:4) blends according to MDI content.

DSC was performed to determine the melting temperature of PLA/PBS blends with different MDI contents. Figure 8a shows a DSC curve for a PLA:PBS blend ratio of 7:3 at varying MDI content, and Figure 8b shows a DSC curve for a PLA:PBS blend ratio of 6:4 at varying MDI content. Under both conditions, two endothermic peaks were observed around 176 °C and 114 °C before MDI was added. As the MDI content was increased, the two endothermic peaks gradually merged into a single peak. Additionally, in both cases, the melting temperature increased slightly as the MDI content increased. This is because the miscibility between PLA and PBS increased as the MDI content increased.

#### 3.3.3. Viscosity Analysis of PLA/PBS Blends According to MDI Content

**Figure 9:**
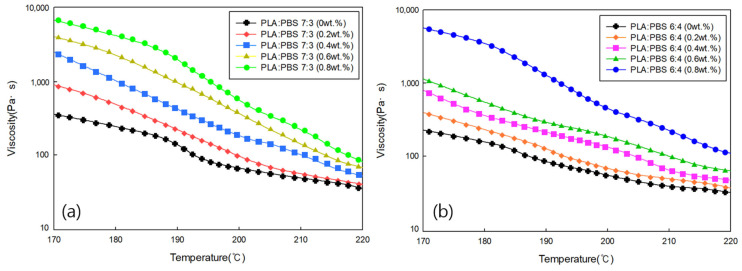
Viscosity of (**a**) PLA/PBS (7:3) and (**b**) PLA/PBS (6:4) blends with different MDI contents.

The rheological properties of PLA/PBS blends at varying MDI content were determined by measuring the viscosity as a function of temperature. Figure 9a shows the viscosity for a PLA:PBS blend ratio of 7:3 at varying MDI content, and Figure 9b shows the viscosity for a PLA:PBS blend ratio of 6:4 at varying MDI content. Under both conditions, the viscosity decreased as the temperature increased. The viscosity of the sample without MDI was the lowest, and the viscosity increased as the content of MDI increased. This is because MDI enhances the miscibility between PLA and PBS, resulting in increased viscosity.

#### 3.3.4. Viscoelastic Behavior Analysis of PLA/PBS Blends According to MDI Content

**Figure 10:**
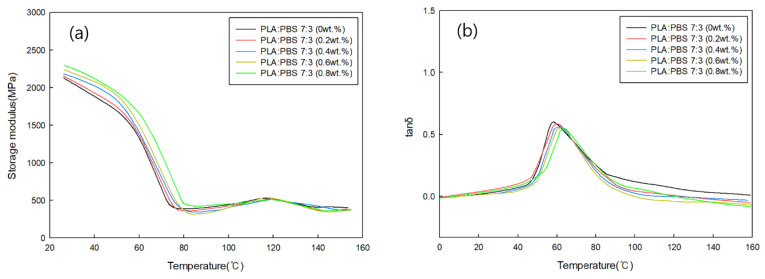
(**a**) Storage modulus and (**b**) tan δ of PLA:PBS (7:3) blends according to MDI content.

**Figure 11:**
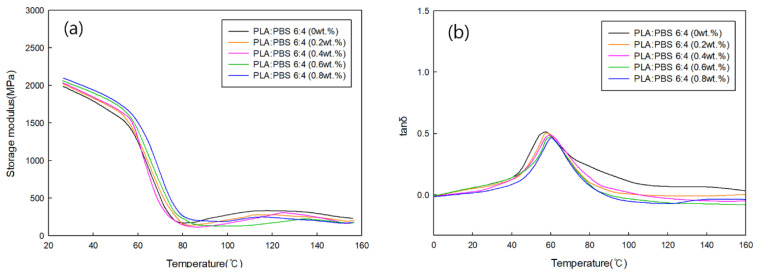
(**a**) Storage modulus and (**b**) tan δ of PLA:PBS (6:4) blends according to MDI content.

DMA was performed to analyze the viscoelastic behavior of PLA/PBS blends with different MDI contents. Figure 10a and Figure 11a display the storage modulus as a function of MDI content for PLA:PBS blend ratios of 7:3 and 6:3, respectively. For both blend ratios, the storage modulus increased with increasing MDI content. This is because MDI improves the miscibility between PLA and PBS, resulting in improved elastic properties. Figure 10b and Figure 11b show tan δ as a function of the MDI content for PLA:PBS blend ratios of 7:3 and 6:3, respectively, from which the glass transition temperature of the specimen was confirmed through the tan δ peak. The tan δ peak showed a slight increase in glass transition temperature as the MDI content increased under all conditions. This is believed to be because the addition of MDI improved the miscibility of PLA and PBS.

#### 3.3.5. Structural Analysis of PLA/PBS Blends According to MDI Content

**Figure 12:**
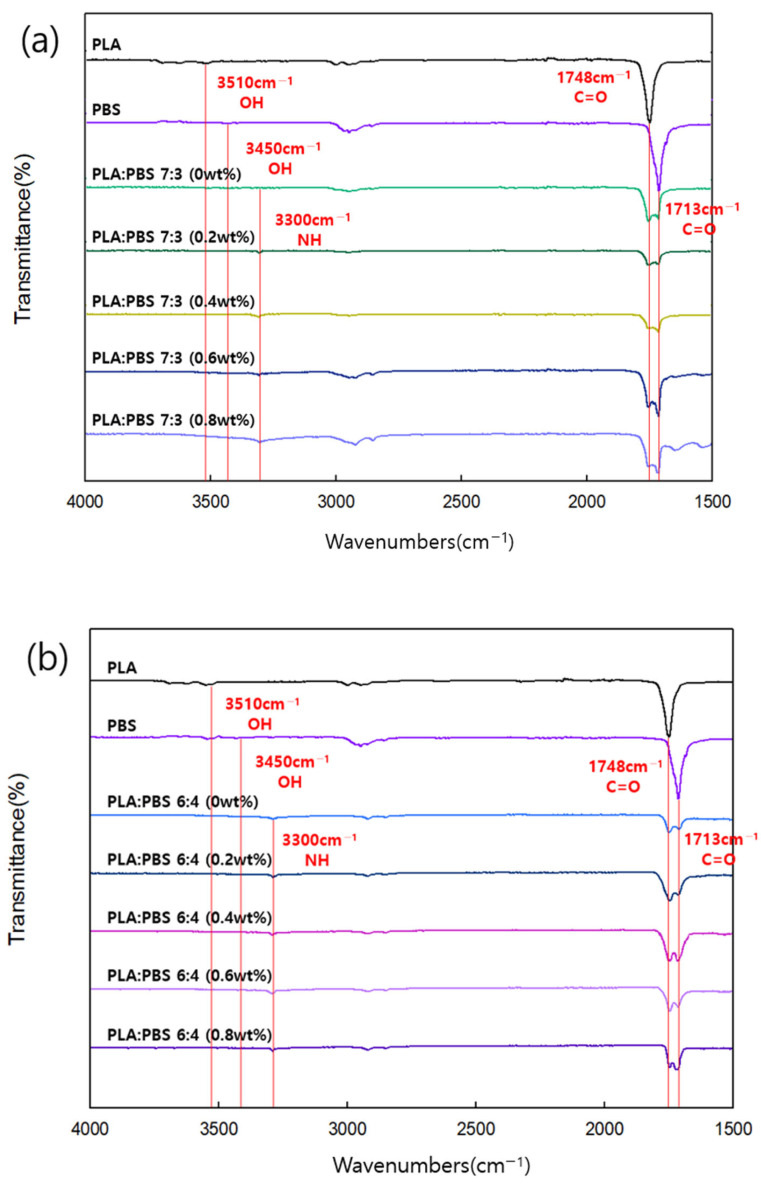
FT-IR of (**a**) PLA/PBS (7:3) and (**b**) PLA/PBS (6:4) blends with varying MDI content.

**Figure 13:**
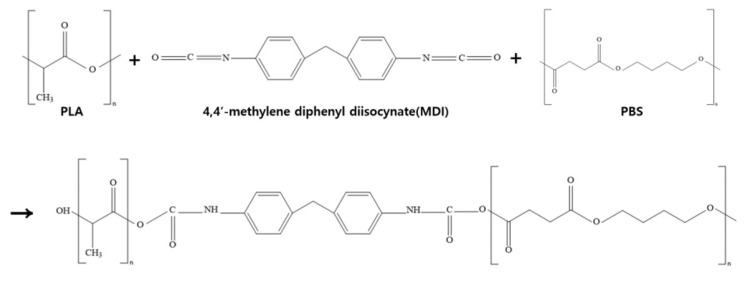
Chemical structure of PLA/PBS blending by MDI.

Figure 12 shows measurements results obtained using FT-IR to confirm the structural change in the PLA/PBS blend with varying MDI content. The structural formula for the chemical bonding of PLA and PBS by MDI is shown in Figure 13. Figure 12a,b show PLA:PBS blend ratios of 7:3 and 6:4, respectively, and in both graphs, PLA showed an -OH- peak at 3510 cm^−1^, whereas PBS showed an -OH- peak at 3450 cm^−1^. However, as MDI was added, the -OH- peak disappeared and a new -NH- peak appeared at 3300 cm^−1^. This is because the chemical bond breaks the double bond of the isocyanate group (NCO group) on both sides of MDI and reacts with the –OH group of PLA and PBS to form an amide group (NHCO group), thereby improving the miscibility between the two polymers. In addition to the changes observed in the –OH and –NH regions, a noticeable variation was also observed in the carbonyl stretching region near 1713 cm^−1^ with increasing MDI content. The absorption band at approximately 1748 cm^−1^ is mainly attributed to the ester carbonyl (C=O) stretching vibration of PLA, whereas the band around 1713 cm^−1^ is associated with the ester carbonyl stretching vibration of PBS. As the MDI content increases, the formation of NHCO-containing linkages and enhanced interfacial interactions between PLA and PBS can alter the local chemical environment of the PBS carbonyl groups. These changes, including increased intermolecular interactions such as hydrogen bonding between N–H and C=O groups, can lead to variations in the intensity and shape of the carbonyl band near 1713 cm^−1^. This behavior provides additional evidence of improved compatibility between PLA and PBS induced by MDI addition.

### 3.4. Characteristics of Manufactured Fibers According to the MDI Content of PLA/PBS Blends

#### 3.4.1. Tensile Strength and Elongation at Break of Blend Fibers According to the MDI Content of PLA/PBS Blends

**Figure 14:**
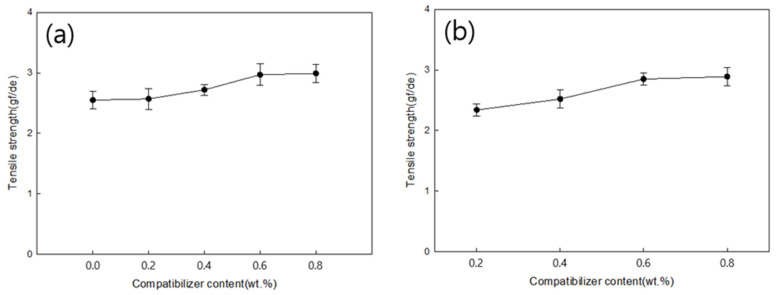
Tensile strength of (**a**) PLA/PBS (7:3) and (**b**) PLA/PBS (6:4) blend fibers according to MDI content.

Figure 14a,b show the tensile strength as a function pf the MDI content for PLA:PBS blend ratios of 7:3 and 6:4, respectively. For blend fibers with a PLA/PBS blend ratio of 7:3, the tensile strength of the blend fiber without MDI was 2.55 gf/de, whereas the addition of 0.8 wt.% MDI increased the tensile strength by approximately 17.25% to 2.99 gf/de. For a PLA/PBS blend ratio of 6:4, the blend without MDI was not spun, but the tensile strength of the blend fiber with 0.2 wt.% MDI was 2.34 gf/de, whereas that of the blend fiber with 0.8 wt.% MDI increased by approximately 23.08% to 2.88 gf/de. Under both conditions, the tensile strength of the blend fibers decreased, compared to neat-PLA fibers. However, as the MDI content increased, the tensile strength of the blend fibers increased. Furthermore, the tensile strength increased more significantly at a PLA/PBS blend ratio of 7:3 than at 6:4. This increase in tensile strength was attributed to the increased miscibility of PLA and PBS due to the addition of MDI.

**Figure 15:**
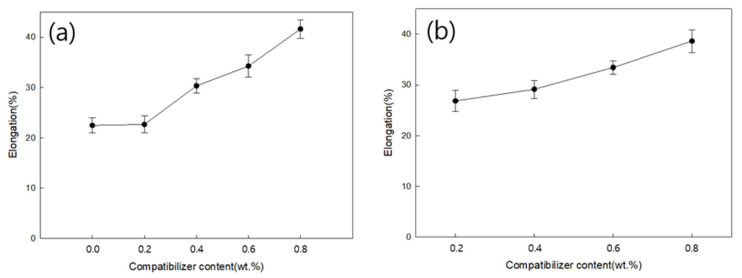
Elongation at break of (**a**) PLA/PBS (7:3) and (**b**) PLA/PBS (6:4) blend fibers according to MDI content.

Figure 15a,b show the elongation at break according to the MDI content for PLA:PBS blend ratios of 7:3 and 6:4, respectively. For blended fibers with a PLA/PBS blend ratio of 7:3, the elongation at break of the blend fiber without MDI was 22.48%, whereas that of the blend fiber with 0.8 wt.% MDI increased by approximately 85.23% to 41.64%. For blended fibers with a PLA/PBS blend ratio of 6:4, the blend without MDI was not spun, but the elongation at break of the blend fiber with 0.2 wt.% MDI was 26.84%, and the tensile strength of the blend fibers with 0.8 wt.% MDI increased by approximately 44.00% to 38.65%. Under both conditions, elongation at break improved substantially with increasing MDI content. The elongation at break was greater at a PLA/PBS blend ratio of 7:3 than at 6:4. This is because the MDI increased the miscibility of PLA and PBS, thereby improving the brittleness of PLA.

#### 3.4.2. Cross-Section of Blend Fibers According to the MDI Content of PLA/PBS Blends

**Figure 16:**
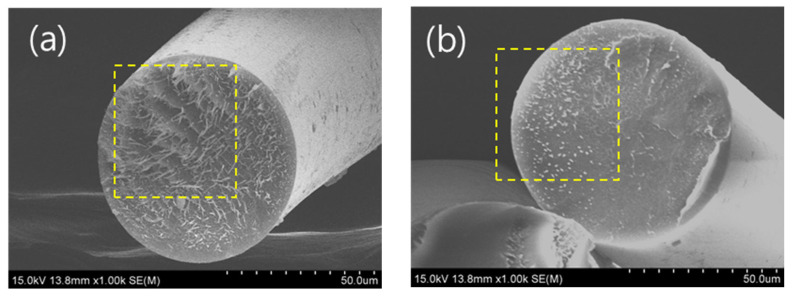

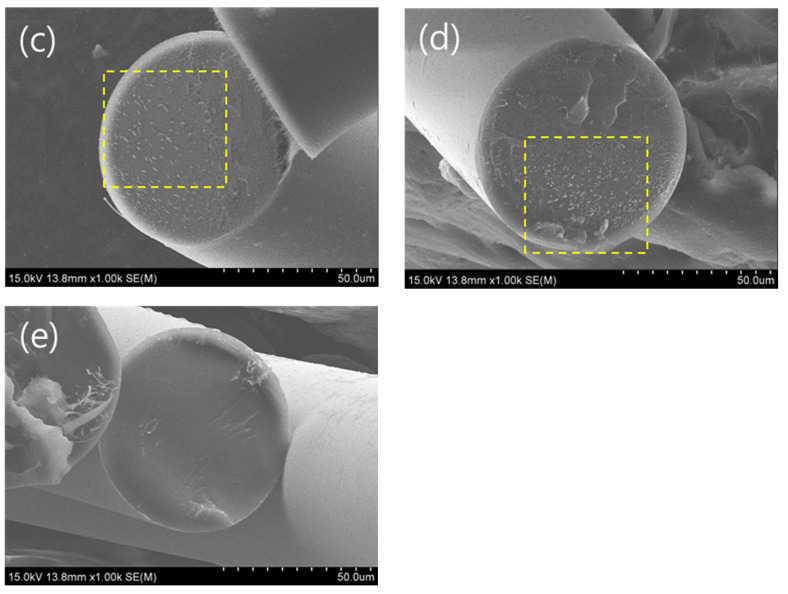
SEM image of PLA:PBS (7:3) blend fibers: (**a**) 0 wt.%, (**b**) 0.2 wt.%, (**c**) 0.4 wt.%, (**d**) 0.6 wt.%, (**e**) 0.8 wt.%.

SEM was used to observe the cross-sections of blend fibers manufactured at a PLA:PBS ratio of 7:3 with varying MDI content. Figure 16a shows the cross-section of a blend fiber without MDI, and with Figure 16b 0.2 wt.% MDI, Figure 16c 0.4 wt.% MDI, Figure 16d 0.6 wt.% MDI, and Figure 16e 0.8 wt.% MDI. As shown in the square yellow marks in Figure 16, phase separation between PLA and PBS was observed in the blend fibers without MDI, and this phase separation gradually alleviated as the MDI content increased. This is attributed to the improved miscibility of PLA and PBS due to the addition of MDI.

## 4. Conclusions

In this study, PLA was blended with poly(butylene succinate) (PBS) to improve the brittleness of PLA. Blends were prepared with varying PBS contents of 0, 10, 20, 30, and 40 wt.%. To improve the miscibility of PLA/PBS blends, MDI was added at varying concentrations of 0, 0.2, 0.4, 0.6, and 0.8 wt.%. The blends were melt-spun, and their properties were analyzed. The findings of this study are summarized as follows:

- As the PBS content increased, the thermal stability, viscosity, elasticity, and glass transition temperature of the blends decreased. In other words, the miscibility of the blends decreased as the PBS content increased.
- As the MDI content in the PLA/PBS blend increased, the thermal stability, viscosity, elastic properties, and glass transition temperature increased. This indicates that the miscibility of the PLA/PBS blend improves with increasing MDI content.
- The tensile strength of the manufactured blend fibers decreased with increasing PBS content. However, the tensile strength and elongation at break significantly improved with increasing MDI content. This is evident in the cross-sectional SEM images of the blend fibers, indicating that phase separation was alleviated.

Therefore, this study demonstrated that when the PBS content in the PLA/PBS blend was 30 wt.%, the tensile strength decreased, but the elongation at break increased significantly. The miscibility of the PLA/PBS blend was highest at 0.8 wt.% MDI, resulting in a significant increase in the tensile strength and elongation at break of the blend fibers.

## Figures and Tables

**Table 1:** Specifications of PLA and PBS.

Specifications	PLA	PBS
Density (g/cm^3^)	1.24	1.26
Tg (°C)	60	−32
Tm (°C)	175	115

**Table 2:** Blend ratio of PLA and PBS.

PLA (wt.%)	100	90	80	70	60
PBS (wt.%)	0	10	20	30	40

**Table 3:** MDI content with respect to the PLA/PBS blend ratio.

PLA/PBS (wt.%)	100	99.8	99.6	99.4	99.2
MDI (wt.%)	0	0.2	0.4	0.6	0.8

**Table 4:** Thermal decomposition onset temperature of PLA/PBS blend ratio.

PLA:PBS	10:0	9:1	8:2	7:3	6:4	0:10
Decomposition onset temperature (°C)	351	356	358	360	363	379

**Table 5:** Thermal decomposition onset temperature of PLA/PBS blends according to MDI contents.

**MDI content (wt.%)**	0.0	0.2	0.4	0.6	0.8
PLA:PBS (7:3)	360	365	367	368	370
PLA:PBS (6:4)	363	366	367	370	371

## Data Availability

The original contributions presented in this study are included in the article. Further inquiries can be directed to the corresponding author.
